# Supplementary material for: An engram of intentionally forgotten information
Source: Nat Commun. 2021 Nov 8;12:6443. doi: 10.1038/s41467-021-26713-x (PMC8575985; doi:10.1038/s41467-021-26713-x)
Supplement: Supplementary file 1 — Supplementary Information [file 41467_2021_26713_MOESM1_ESM.pdf]

Supplementary Information

An engram of intentionally forgotten information

Sanne Ten Oever, Alexander T. Sack, Carina R. Oehrn, Nikolai Axmacher

Supplementary figures

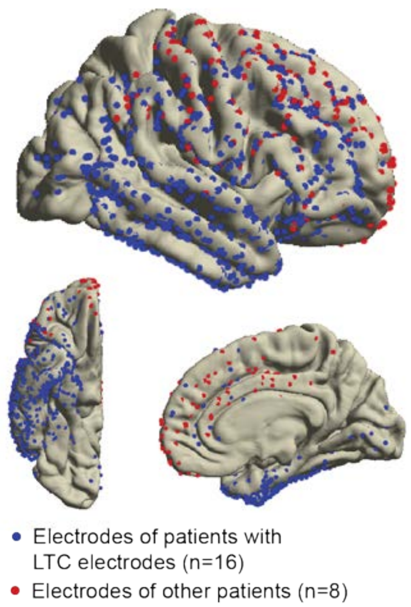

Supplementary figure 1: Electrode positioning of all included patients. Blue channels indicate electrodes of patients with LTC electrodes and red includes all other patients.

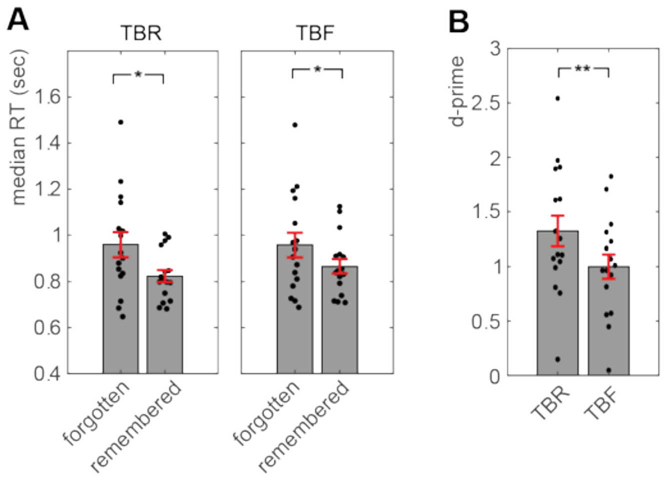

Supplementary figure 2: Additional behavioral analyses (n=16). A) Reaction time effects. B) d-prime effects. Asterisk and double asterisk indicate significance at the alpha 0.05 and 0.01 level, respectively (two-sided paired t-tests). Error bars indicate the standard error of the mean. Source data are provided as a Source Data file.

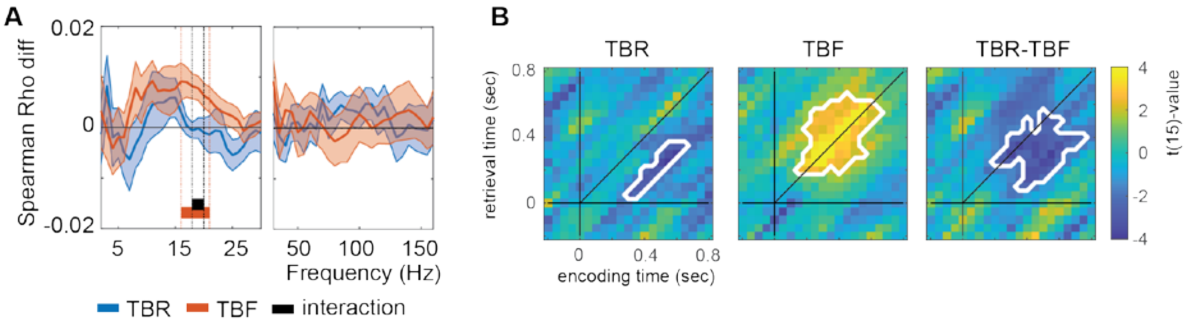

Supplementary figure 3: Additional analyses on encoding-retrieval similarity. A) Encoding-retrieval similarity in the LTC controlling for trial numbers. Conventions are the same as in Figure 3B. Significant effects for the low frequencies were found for the TBF items ($p = 0.047$) and the difference between the TBF and TBR ($p = 0.040$). B) Time-shifting encoding-retrieval similarity analyses using various encoding and retrieval times at the frequency of the significant difference between TBR and TBF items. We found significantly decreased encoding-retrieval similarity for TBR items ($p = 0.045$), increased reactivation for TBF items ($p = 0.006$) and a significant difference ($p=0.005$). Error bars indicate the standard error of the mean. Source data are provided as a Source Data file.

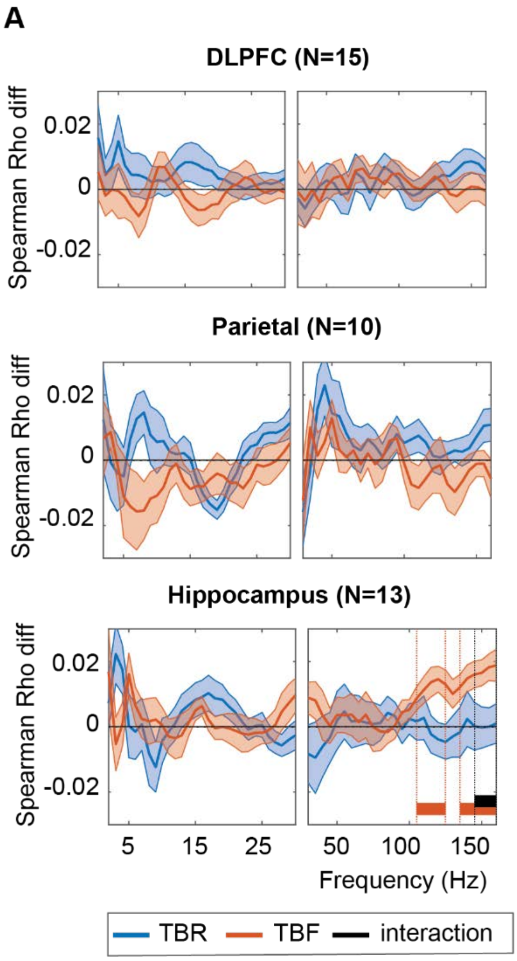

Supplementary figure 4: Encoding-retrieval similarity for different ROIs. Conventions are the same as in Figure 3B. Error bars indicate the standard error of the mean. Source data are provided as a Source Data file.

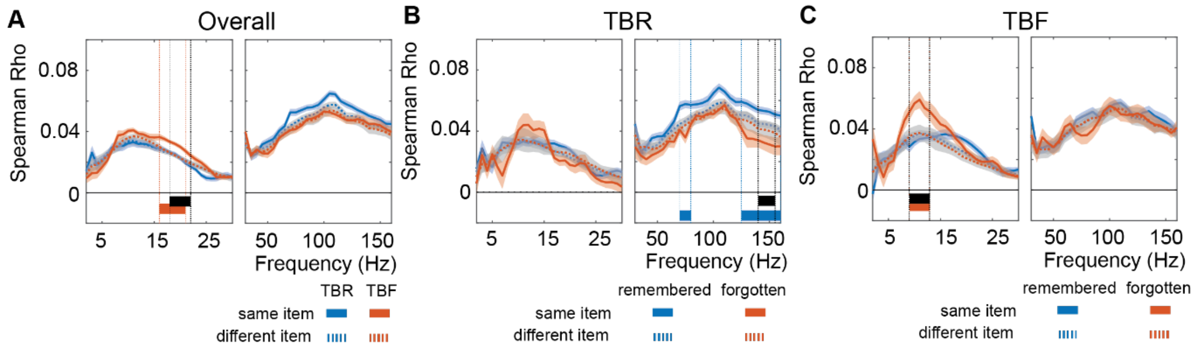

Supplementary figure 5: Raw correlation traces of the encoding-retrieval similarity analysis of same vs. different items. Significance lines reflecting the encoding-retrieval similarity comparisons (on-diagonal vs. off-diagonal) are identical to those shown in the main manuscript. Effects are driven by increases in same-item correlations rather than by decreases in the different-item correlations. Conventions are the same as in Figure 3B. Error bars indicate the standard error of the mean. Source data are provided as a Source Data file.

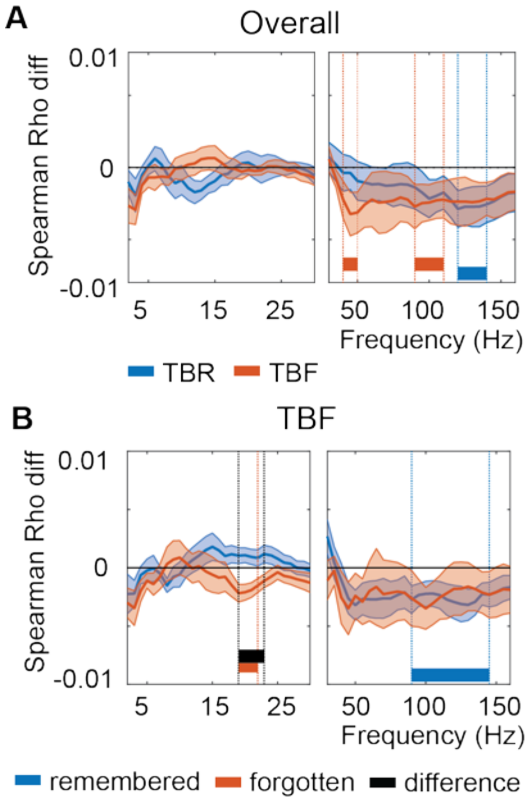

Supplementary figure 6. Comparing off-diagonal encoding-retrieval similarity with encoding-retrieval similarity of novel items. A) To elaborate that our effects are not due to non-specific effects to novel items, we compared the similarities between TBF/TBR items during encoding with either other TBF/TBR items during retrieval (excluding same encoding-retrieval pairs) or with novel items, e.g., we contrasted different-item same-class encoding-retrieval similarity versus novel encoding-retrieval similarity. We found that for TBR/TBF items, correlations were significantly lower within their class (e.g. encoding TBR – retrieval TBR) than with novel items (e.g. encoding TBR – retrieval novel; TBR: 120-140 Hz; $p=0.029$; min $t(15)$-value = -2.21; TBF cluster 1: 90-110 Hz; $p = 0.020$; min $t(15)$-value = -1.87; TBF cluster 2: 40-50 Hz; $p= 0.047$; min $t(15)$-value = -2.17). No differences were found between the TBR and TBF conditions. B) When we split up the analysis for the remembered and forgotten TBF items, we again found stronger similarities for different-item same-class correlations versus encoding-retrieval similarity for novel items in a high frequency window for remembered TBF items (90-145 Hz; $p = 0.004$; min $t(15)$-value = -25.79). Interestingly, we found the same reduced between-item same-class similarities compared to novel items for forgotten TBF items (19-22 Hz; $p = 0.038$; min $t(15)$-value = -

2.56). There was also a significant difference between forgotten compared to remembered TBF items (19-23 Hz; $p = 0.023$; max t(15)-value = 2.87). This suggests that encoding items are overall more similar to novel items during retrieval. Since novel items have not been seen before during the task, perceiving a novel item may induce an 'encoding' signal. This is in contrast with a 'retrieval' signal, which should contain the information that a particular item has been presented before. Thus, it seems that activity during presentation of novel items is more similar to the encoding signal of forgotten TBF items compared to the retrieval TBF signals (also see Supplementary Figure 7). Blue and orange bars indicate significant item-specific encoding-retrieval similarity of remembered and forgotten items respectively. Shaded error bars indicate the standard error of the mean. Source data are provided as a Source Data file.

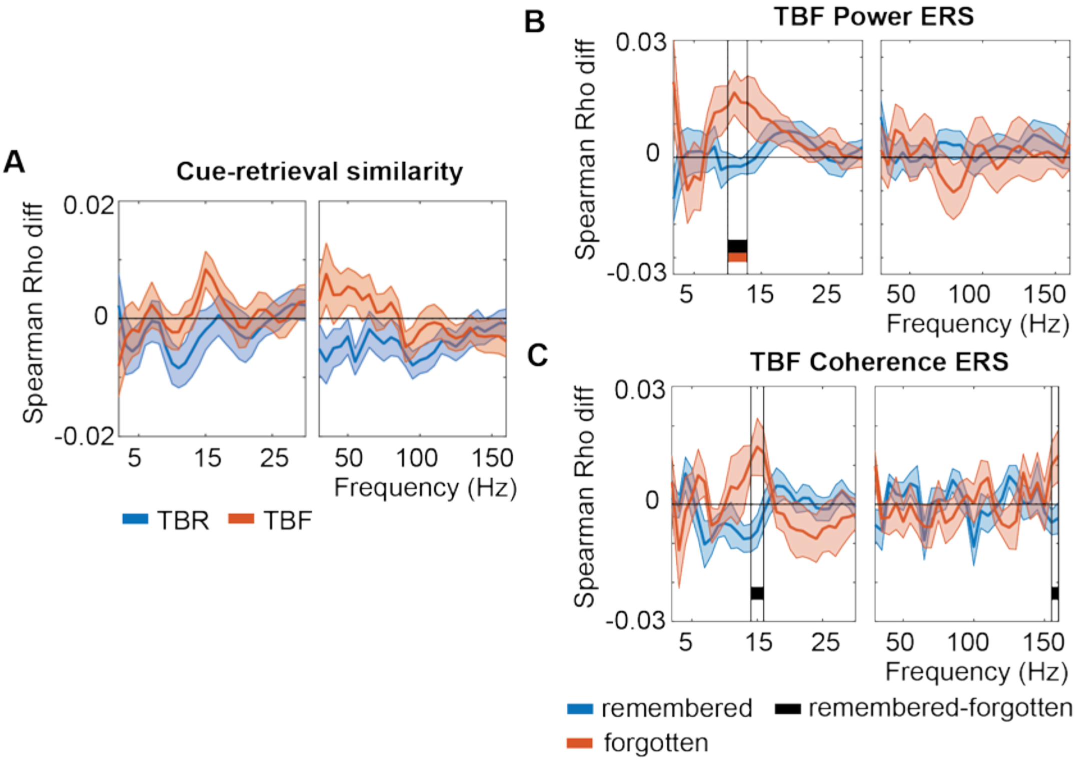

Supplementary figure 7. Instruction-specific cue-retrieval similarity and cue-corrected encoding-retrieval similarity. A) We correlated activity during the forgetting instruction of one item with activity during the presentation of a different TBF item during retrieval. The same was done for activity during remembering instructions and presentation of different TBR items during retrieval, and correlations were then compared between conditions. This analysis of instruction-specific cue-retrieval similarity is relevant for the question of context reactivation, because the forgetting context is shared among all TBF items, but does not occur for the TBR items. We found no difference between same- or different-instruction cue-retrieval similarity. The figure displays the difference between same-instruction cue-retrieval similarity and different-instruction cue-retrieval similarity (legend indicates the instruction at the cue). B) Encoding-retrieval similarity as in Figure 4A, but using partial correlations correcting for cue activity (at matched timepoints and frequencies). The differences between TBF remembered and TBF forgotten trials as well as the contrast between same versus different TBF forgotten items remained significant ($p = 0.043$ and $p = 0.041$, respectively). C) Encoding-retrieval similarity as in Figure 5A, but using partial correlations correcting for cue activity. The difference between remembered versus forgotten TBF trials remained significant ($p = 0.03$). The contrast between same

versus different TBF forgotten items appeared qualitatively similar, but did not reach significance (largest cluster: p = 0.197). Shaded error bars indicate the standard error of the main. Source data are provided as a Source Data file.

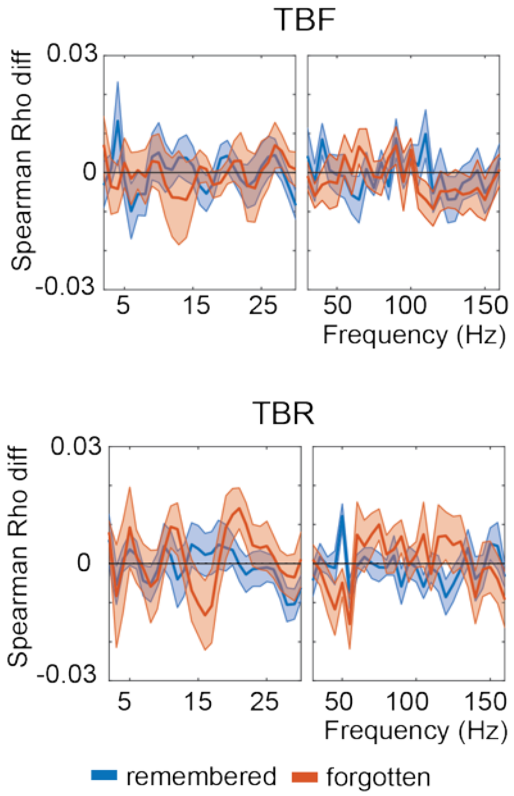

Supplementary figure 8. Hippocampus-LTC coherence effects. Conventions are the same as in Figure 3B. Error bars indicate the standard error of the mean. Source data are provided as a Source Data file.

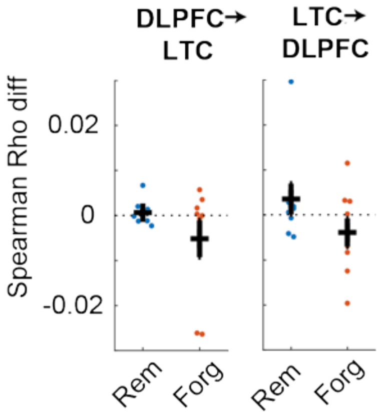

Supplementary figure 9: Alpha/beta transfer entropy results for to-be-remembered items (n=10). Conventions are the same as in Figure 6A. Error bars indicate the standard error of the mean. Significance was tested by two-sided paired t-tests. Source data are provided as a Source Data file.

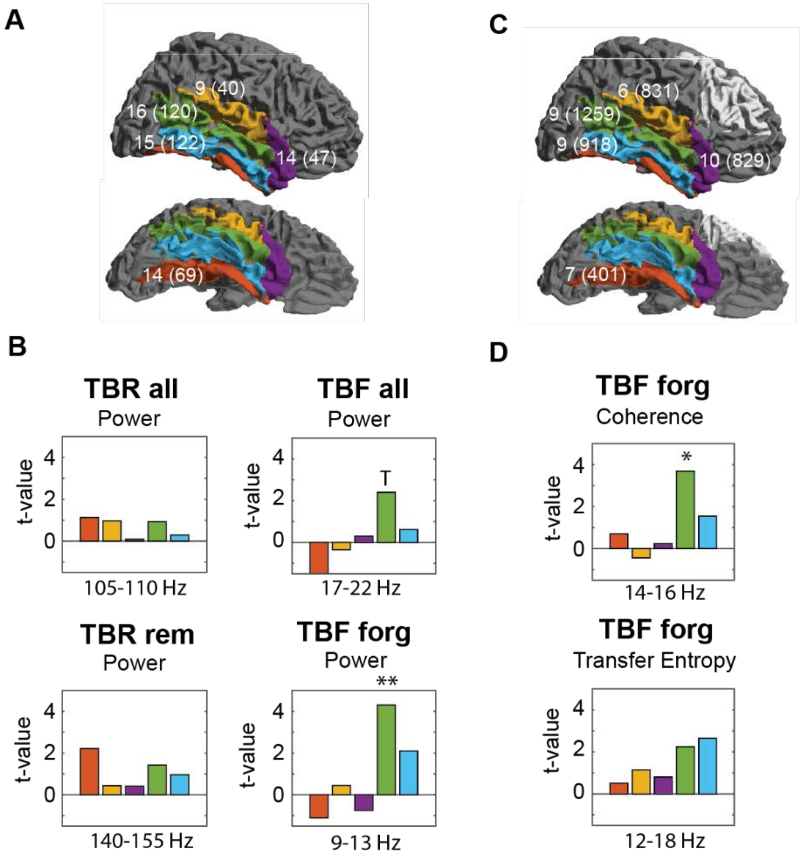

Supplementary figure 10: Regional distribution of encoding-retrieval similarity in the time window from 0.1-0.5s. A) Lateral temporal cortex (LTC) subregions in which numbers indicate the number of participants and channels (in parentheses) included in the analyses. B) Encoding-retrieval similarity based on power at 105-110 Hz for TBR (top-left), 140-155 Hz for TBR remembered items (bottom-left), and 17-22 Hz for TBF (top-right) and 9-13 Hz for TBF forgotten items (bottom-right). C) LTC subregions in patients with additional DLPFC electrodes. Numbers indicate the number of participants and channel pairs (in brackets) that were included in the analyses. D) Encoding-retrieval similarity of TBF forgotten items based on coherence at 14-16 Hz (top) and based on transfer entropy bandpass filtered between 12-18 Hz (bottom). Results were Bonferroni corrected for multiple comparisons in the 5 subregions. Single and double asterisks indicate significance at $p<0.05$ and $p<0.01$ and T represents a trend ($p<0.10$). Significance was tested by one-sided paired t-tests. (Power TBF all trend $p = 0.074$; power TBF forgotten $p = 0.002$; Coherence $p = 0.015$). Source data are provided as a Source Data file.

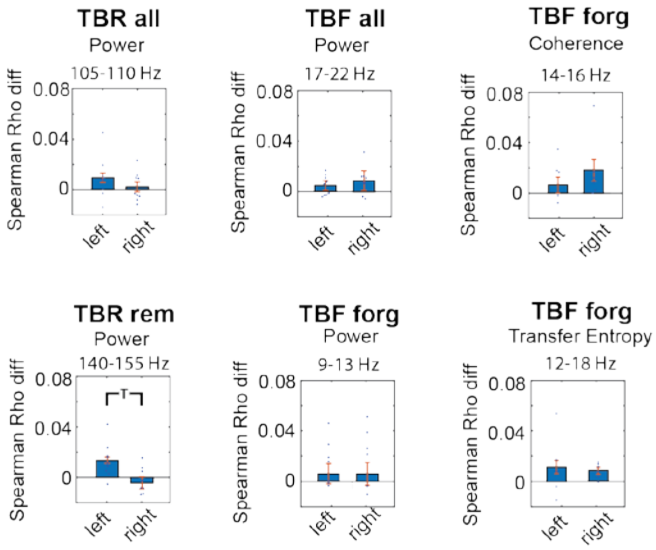

Supplementary figure 11: Hemisphere-specific effects in the time window from 0.1-0.5s (power: n=9. Coherence: n = 6. Only subjects with electrodes on both sides are included). All comparisons are based on the same frequency ranges as reported in Supplementary Figure 10. T indicates trend significant effect (p <0.10). Error bars indicate the standard error of the mean. Test pertain a two-sided paired t-test (p = 0.001). Source data are provided as a Source Data file.

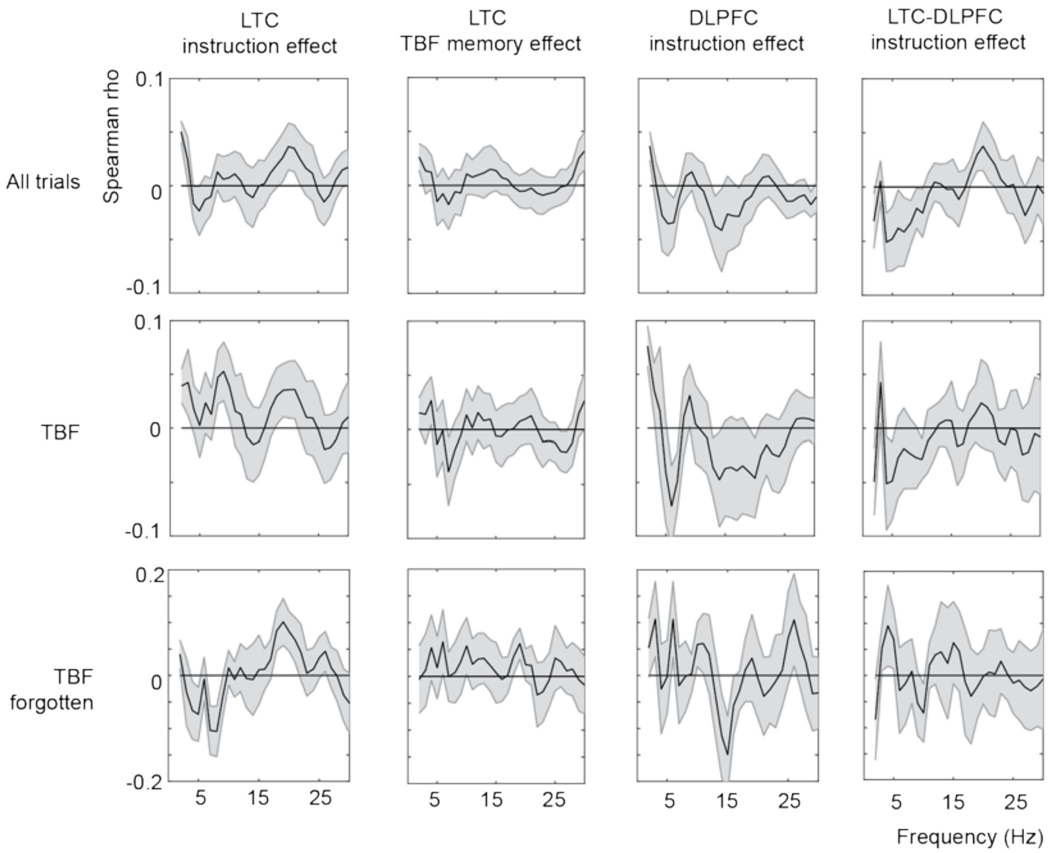

Supplementary figure 12: Intra-individual correlations between single trial cue power/coherence and single trial ERS values. Correlations are estimated at the frequency ranges of the cue effects (see Figure 7) and using either all trials, only TBF trials, or only successfully forgotten TBF trials. Error bars indicate the standard error of the mean. Source data are provided as a Source Data file.
