# Supplementary material for: An engram of intentionally forgotten information
Source: Nat Commun. 2021 Nov 8;12:6443. doi: 10.1038/s41467-021-26713-x (PMC8575985; doi:10.1038/s41467-021-26713-x)
Supplement: Supplementary file 3 — Reporting Summary [file 41467_2021_26713_MOESM3_ESM.pdf]

nature research

Corresponding author(s): Sanne ten Oever
Last updated by author(s): Sep 20, 2021

Reporting Summary

Nature Research wishes to improve the reproducibility of the work that we publish. This form provides structure for consistency and transparency in reporting. For further information on Nature Research policies, see our Editorial Policies and the Editorial Policy Checklist.

Statistics

For all statistical analyses, confirm that the following items are present in the figure legend, table legend, main text, or Methods section.

n/a	Confirmed	
☐	☒	The exact sample size (n) for each experimental group/condition, given as a discrete number and unit of measurement
☐	☒	A statement on whether measurements were taken from distinct samples or whether the same sample was measured repeatedly
☐	☒	The statistical test(s) used AND whether they are one- or two-sided<br>*Only common tests should be described solely by name; describe more complex techniques in the Methods section.*
☐	☒	A description of all covariates tested
☐	☒	A description of any assumptions or corrections, such as tests of normality and adjustment for multiple comparisons
☐	☒	A full description of the statistical parameters including central tendency (e.g. means) or other basic estimates (e.g. regression coefficient) AND variation (e.g. standard deviation) or associated estimates of uncertainty (e.g. confidence intervals)
☐	☒	For null hypothesis testing, the test statistic (e.g. F, t, r) with confidence intervals, effect sizes, degrees of freedom and P value noted<br>*Give P values as exact values whenever suitable.*
☒	☐	For Bayesian analysis, information on the choice of priors and Markov chain Monte Carlo settings
☒	☐	For hierarchical and complex designs, identification of the appropriate level for tests and full reporting of outcomes
☐	☒	Estimates of effect sizes (e.g. Cohen's d, Pearson's r), indicating how they were calculated

Our web collection on statistics for biologists contains articles on many of the points above.

Software and code

Policy information about availability of computer code

Data collection	Electrophysiological data were recorded with the digital EPAS system (Schwarzer, Munich, Germany) and its implemented Harmonie EEG software v7.0a (Stellate, Quebec, Canada).
Data analysis	Matlab v.2016a; Freesurfer v.6; Fieldtrip v.20161231; Circular Toolbox 2012a; GCMI toolbox v.0.3

For manuscripts utilizing custom algorithms or software that are central to the research but not yet described in published literature, software must be made available to editors and reviewers. We strongly encourage code deposition in a community repository (e.g. GitHub). See the Nature Research guidelines for submitting code & software for further information.

Data

Policy information about availability of data

All manuscripts must include a data availability statement. This statement should provide the following information, where applicable:

- Accession codes, unique identifiers, or web links for publicly available datasets
- A list of figures that have associated raw data
- A description of any restrictions on data availability

Data will be made available upon request to sanne.tenoever@mpi.nl or nikolai.axmacher@ruhr-uni-bochum.de

Field-specific reporting

Please select the one below that is the best fit for your research. If you are not sure, read the appropriate sections before making your selection.

☒ Life sciences ☐ Behavioural & social sciences ☐ Ecological, evolutionary & environmental sciences

For a reference copy of the document with all sections, see nature.com/documents/nr-reporting-summary-flat.pdf

Life sciences study design

All studies must disclose on these points even when the disclosure is negative.

Sample size	The data was collected for a previous study, so for the current design no sample size determination was performed. Based on sample sizes and effect sizes of previous iEEG studies (see e.g. Oehrn et al., 2018; Pacheco-Estefan et al., 2019) a sample size of 16 (patients with lateral temporal electrodes) is sufficient.
Data exclusions	We had to exclude 12 patients because of missing data (either in the EEG or in the post MR implantation scheme). Six more patients were excluded due to poor behavioral performance (more false alarms than hits in the to-be-remembered condition and/or fewer than 20% remembered to-be-rememberd items).
Replication	Several control analyses are performed that showed similar patterns as the original findings. These are reported in the supplementary analyses (a total of 4 different analyses pertaining the same effects). No direct replication in an independent cohort is reported.
Randomization	All patients went through all conditions, so there was no need for randomization in experimental groups. The trial types within a session were randomized per participant.
Blinding	We did not have different experimental groups, so blinding was not relevant for the current study.

Reporting for specific materials, systems and methods

We require information from authors about some types of materials, experimental systems and methods used in many studies. Here, indicate whether each material, system or method listed is relevant to your study. If you are not sure if a list item applies to your research, read the appropriate section before selecting a response.

Materials & experimental systems

n/a	Involved in the study	
☒	☐	Antibodies
☒	☐	Eukaryotic cell lines
☒	☐	Palaeontology and archaeology
☒	☐	Animals and other organisms
☐	☒	Human research participants
☒	☐	Clinical data
☒	☐	Dual use research of concern

Methods

n/a	Involved in the study	
☒	☐	ChIP-seq
☒	☐	Flow cytometry
☐	☒	MRI-based neuroimaging

Human research participants

Policy information about studies involving human research participants

Population characteristics	From the patients with lateral temporal electrodes (N=16), 8 were female. The mean age was 41.3 (standard deviation of 14.9). All patients had with pharmacoresistant epilepsy who had been implanted with intracranial EEG electrodes for diagnostic purposes.
Recruitment	This study involves a study with patients with pharmacoresistant epilepsy who had been implanted with intracranial EEG electrodes for diagnostic purposes. Patients that were planned for this surgery at the Department of Epileptology were requested to participated in the study. As this recordings pertain patient data, results should be interpreted with caution as this involves a patient group and brain activity could be deviating from normal. Note however that we only included electrodes which were free of morphological alterations identified via MRI and were outside of the seizure onset zone.
Ethics oversight	The study was approved by the ethics committee of the University of Bonn.

Note that full information on the approval of the study protocol must also be provided in the manuscript.

Magnetic resonance imaging

Experimental design

Design type	Only anatomical MRIs were collected
Design specifications	Only anatomical MRIs were collected
Behavioral performance measures	Only anatomical MRIs were collected

Acquisition

Imaging type(s)	Structure MRI
Field strength	3.0 T
Sequence & imaging parameters	The sequencing parameters were clinically defined and varied per patient. Mostly a whole brain scan was used with a Gradient Echo at 1 mm isotropic, with a repetition time of 8.16 ms, echo time of 3.74 ms, flip angle of 8 degrees, and a matrix size of 256*256. Patient position was head First-Supine.
Area of acquisition	Whole brain scan

Diffusion MRI ☐ Used ☒ Not used

Preprocessing

Preprocessing software	FreeSurfer v6 and Fieldtrip20161231
Normalization	Normalization was performed in Fieldtrip (which uses the SPM12 implementation). We use a nonlinear normalization.
Normalization template	MNI305
Noise and artifact removal	To ensure alignment of the electrodes and brain we used the hull method introduced by Dykstra to back-project the ECoG electrodes onto the cortical surface. This method is implemented in Fieldtrip v20161231
Volume censoring	Cerebellum was removed from the MRI (using the segmentation of FreeSurfer v.6)

Statistical modeling & inference

Model type and settings	No statistics on the MRI
Effect(s) tested	No statistics on the MRI

Specify type of analysis: ☒ Whole brain ☐ ROI-based ☐ Both

Statistic type for inference (See Eklund et al. 2016)	No statistics on the MRI
Correction	No statistics on the MRI

Models & analysis

n/a	Involved in the study
☒	☐ Functional and/or effective connectivity
☒	☐ Graph analysis
☒	☐ Multivariate modeling or predictive analysis
